# Association of daily perceived stress with fertility outcomes in natural and controlled ovarian stimulation cycles: a feasibility study

**DOI:** 10.1007/s10815-026-03972-5

**Published:** 2026-07-24

**Authors:** Harmonie Strohl, Nguyen Do, Howon Ryu, Olumayowa Dayo, Emily Ho, Jacey Hu, Alexander Kauffman, Ina Stelzer, Ariel Lang, Kellie Church, Jingjing Zou, Irene Su

**Affiliations:** 1 School of Medicine, University of California, La Jolla, San Diego USA; 2 Herbert Wertheim School of Public Health and Human Longevity Science, University of California, La Jolla, San Diego USA; 3 Department of Obstetrics, Gynecology, and Reproductive Sciences, University of California, La Jolla, San Diego USA; 4 Obstetrics and Gynecology Institute, Cleveland Clinic Foundation, Cleveland, USA; 5 Department of Psychiatry, University of California, La Jolla, San Diego USA; 6 Center of Excellence for Stress and Mental Health, VA San Diego Healthcare System, San Diego, USA; 7 Moores Cancer Center, University of California, La Jolla, San Diego USA

**Keywords:** Perceived stress, Infertility, Ovulation, Luteinizing hormone, In vitro fertilization, Ecologic momentary assessment

## Abstract

**Purpose:**

Females with infertility perceive stress, but most studies rely on single assessments. We examined the feasibility of repeated ecological momentary assessment (EMA) during IVF and whether stress trajectories across two cycles were associated with reproductive and IVF outcomes.

**Methods:**

In a prospective cohort at a single academic center, 26 females (18–42 years) undergoing IVF with luteal estradiol priming were recruited; 25 completed follow-up. Perceived stress was assessed via text-message EMA three times daily during a priming month and the subsequent controlled ovarian stimulation (COS) cycle. Primary outcomes were ovulation in the priming cycle and a composite poor COS outcome (cycle cancellation, < 4 oocytes retrieved, no blastocyst, or no euploid blastocyst). Functional principal components analysis characterized stress trajectories, and regression models tested associations with outcomes.

**Results:**

Mean age was 36 (SD 3.6); 64% were White and 24% Hispanic. All participants ovulated during priming. Two stress patterns explained 96% of follicular-phase variability. The dominant pattern (early decline then plateau) was not associated with outcomes. A second pattern (peak around cycle day 6 with subsequent rise) was associated with lower peak LH (b − 2.85, 95% CI − 4.60 to − 1.06; adjusted *p* = 0.004). Ten participants (40%) met the composite poor COS outcome, which was not associated with either stress pattern. Stress levels were similar between priming and COS.

**Conclusion:**

EMA is feasible during IVF. In patients reporting mild-to-moderate stress, perceived stress was stable and not associated with key IVF outcomes, though specific trajectories may modestly relate to hypothalamic-pituitary-ovarian-axis function.

## Introduction

Infertility affects approximately 1 in 6 individuals [1–3]. Females with infertility often experience stress from their condition [4, 5]. In response to extreme stress, high corticosteroid-releasing hormone (CRH) and glucocorticoid levels suppress the hypothalamic-pituitary-ovarian (HPO) axis leading to anovulation, which overtly presents as irregular or absent menses and infertility [6, 7]. For females with infertility who report regular menses, the effect of stress on fertility is less clear. Perceived stress refers to how an individual experiences stress, i.e., appraises situations and selects coping abilities, rather than the stress event itself [8]. Interest in measuring perceived stress stems from strong correlations with mental health outcomes including anxiety, depression, and harmful behaviors.

Observational studies suggest that up to one-third of cycles in females in the general population reporting regular menses lack evidence of ovulation as measured by post-ovulatory progesterone, suggesting that sporadic anovulation may be a common, intermittent occurrence [9]. Secondary analysis of the EAGeR trial of females with prior pregnancy loss but no infertility found that participants in the highest perceived stress quartile had lower estrogen and progesterone levels across the menstrual cycle, a 28% higher risk of anovulation, and longer time to pregnancy compared to participants in the lowest stress quartile [10]. Similar ovarian function and sporadic anovulation data in infertile females with regular menses are lacking.

Females who undergo in vitro fertilization (IVF) can serve as a model for studying the association between patterns of perceived stress and menstrual cycle and IVF outcomes. IVF cycles that utilize luteal phase priming protocols occur over two consecutive months [11]. In the priming month, individuals undergo an unmedicated cycle until exogenous estradiol is begun after ovulation, enabling studying the association of stress with follicular phase length and ovulation as outcomes of a natural/physiologic cycle. In the following month, individuals undergo controlled ovarian stimulation (COS) with the goal of oocyte retrieval and embryo generation, enabling studying the association of stress with IVF outcomes.

Pre-clinical studies suggest that timing of stress across the follicular phase may differentially impact surge versus pulsatile LH release. Stress occurring during the early- to mid-follicular phase may preferentially reduce activity of KNDy neurons and decrease pulsatile release of GnRH and LH to prolong the follicular phase [12]. Stress occurring during the late follicular phase may negatively regulate AVPV kisspeptin neurons, thus inhibiting GnRH and LH surges and preventing or delaying ovulation [13]. Timing of stress on ovarian function outcomes has not been explored in females with infertility undergoing IVF.

Advances in technology-enabled, real-time ecological momentary assessment (EMA) allow for higher resolution and more accurate phenotyping of perceived stress in IVF [14]. Several studies outside of infertility support the validity of perceived stress measured via EMA by demonstrating positive associations to biomarkers of stress including salivary cortisol, blood pressure, and heart rate [15]. Approximately half of EMA studies report direct, within-person associations between moments of higher perceived stress and concurrent elevations in cortisol and cardiovascular indicators [15]. EMA studies on perceived stress in infertility patients could facilitate more accurate phenotyping and the opportunity to explore if timing of stress in the follicular phase impacts fertility outcomes.

Using EMA to track perceived stress, our feasibility study aimed to characterize the pattern of stress across an IVF cycle and assess whether stress would be associated with HPO function and COS outcomes in females with infertility undergoing IVF. We hypothesized that increased psychosocial stress is associated with anovulation, longer follicular phase length, and worse IVF outcomes. We secondarily explored if outcomes would vary by stress experienced in the early-, mid-, or late-follicular phase.

## Materials and methods

The study was a prospective cohort study of females undergoing IVF at a single academic center. Females were eligible if they were ages 18 to 42, reported regular menstrual cycles between 25 and 35 days over the prior year, had a uterus, did not have exposure to sex steroid hormones within 3 months, planned to undergo IVF using the luteal priming protocol, and used an Apple iPhone (platform for urine hormone measurement using the Inito Fertility Monitor™). The luteal priming protocol is the default protocol for all patients in the center with regular menstrual cycles and normal ovarian reserve. Females with PMOS, functional hypothalamic amenorrhea, BMI < 18.5, eating disorders, decreased ovarian reserve (fewer than six antral follicles or follicle stimulating hormone (FSH) ≥ 11 IU/L), or signs of early menopausal transition (menstrual cycle length difference of more than 7 days from peak reproductive age) [16], were not eligible. Eligible individuals underwent the informed consent process. Consented individuals received the Inito Fertility Monitor™ test strips and up to $75 for participation. The study was approved by the University of California, San Diego Institutional Review Board.

**Fig. 1:**
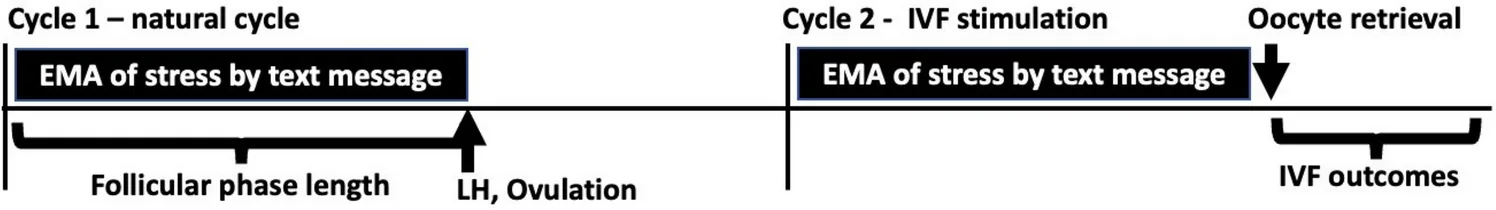
Cohort design indicating ecological momentary assessment of perceived stress by three-times-daily text messaging and outcomes over a natural cycle and a controlled ovarian stimulation cycle

Each participant contributed data from one IVF cycle that occurred over a priming month and a subsequent COS month (Fig. 1). During the priming month, participants were instructed to use the second urination of the morning to test for the LH surge using the Inito device. Cycle day 1 was defined as the first day of menstruation; participants tested for the LH surge each morning, starting on cycle day 6 until ovulation was confirmed. As part of the luteal priming protocol, they began oral estradiol (4 mg daily) 7 days after the LH surge for 14–21 days. Following priming with estradiol, participants underwent GnRH antagonist COS cycles using individualized gonadotropin dosing and dual trigger with leuprolide (4 mg, two doses over 12 h) and hCG (1000 IU for high responders and 10,000 IU for others). Oocyte retrieval occurred 35.5 h post trigger. Mature oocytes underwent intracytoplasmic sperm injection with sequential culture to the blastocyst stage and blastocyst cryopreservation on days 5, 6, or 7 with or without preimplantation genetic testing for aneuploidy.

## Daily measurement of perceived stress

During the follicular phases of the priming (menstrual cycle day 3 to ovulation) and COS (start of stimulation to oocyte retrieval) cycles, participants received text messages to rate their stress three times daily (8am, 2 pm, 8 pm). The text prompt read, “On a scale of 1 (least) to 10 (most), how stressed are you?” During the consent process, patients were informed that 1 referred to not being stressed at all and 10 referred to the most stress they have ever felt. For analyses, stress measurements from cycle day 3 of the priming cycle until day of LH surge and from COS day 1 to the day of the oocyte retrieval were used. We derived daily mean, maximum, and minimum stress level per participant per day.

## Measurement of outcomes

Urinary levels of FSH, LH, estrone 3-glucuronide (E3G), and PdG were measured in daily urine from cycle days 6 through confirmation of ovulation using the Inito device during the priming cycle.

The Inito monitor is a home-use device that uses lateral flow assays and a monitor that mounts onto a smartphone. The monitor utilizes the camera and a controlled light source within the device to measure the intensity of lines on the test strip, thereby deriving LH, E3G, and PdG levels [17]. High correlations between serum and urinary levels in validation studies—estradiol (*R*^2^ = 0.96), progesterone (*R*^2^ = 0.95), and LH (*R*^2^ = 0.98)—support the device as a reliable proxy for serum hormone measurements [18]. Ovulation is defined as ≥ 2.75 × rise in PdG in one urine sample within 3 days after the LH peak, which achieved an area under the curve (AUC) of 0.981. Hormone levels were shared in real-time with investigators using a research dashboard.

For the priming cycle, the primary outcomes were ovulation (yes/no) and length of follicular phase, defined as cycle day 1 of the priming cycle to the day of LH surge. LH levels on the day of the LH surge were the secondary outcome.

For the COS cycle, IVF outcomes were derived from electronic health records. The primary outcome was a composite poor outcome of cycle cancellation, fewer than 4 oocytes retrieved, no blastocyst embryo development, or no euploid blastocyst. Length of stimulation was the secondary outcome. Clinical pregnancy and live birth following frozen embryo transfer were exploratory outcomes.

## Measurement of covariates

At enrollment, participants completed a questionnaire on demographic, medical, and fertility history (Table 1). Depression and anxiety symptoms were assessed using the Patient Health Questionnaire-9 (PHQ-9) and the Generalized Anxiety Disorder-7 (GAD-7), respectively. A cutoff score of ≥ 10 on either scale was used to identify participants who met the criteria for clinically significant depression or anxiety. The Fertility Quality of Life (FertiQOL) scale was also used to assess the impact of fertility problems across four subdomains: Emotional (emotional responses to infertility), Mind–Body (effects on physical and cognitive functioning), Relational (impact on partner relationships), and Social (effects on social support and participation). Scores on the FertiQOL range from 0 to 100, with higher scores indicating better quality of life.

**Table 1:** Baseline and IVF cycle characteristics of participants (*n* = 25)

Mean age (SD)	36.0 (3.6)
30–35 years, *n* (%)	13 (52)
36–40 years	7 (28)
41–42 years	5 (20)
BMI	
Mean (SD)	24.4 (3.1)
18.5–24.9, *n* (%)	15 (60)
25.0–29.9	10 (40)
Race	
White, *n* (%)	16 (64)
Black or African American	1 (4)
Asian	5 (20)
Native Hawaiian or Pacific Islander	1 (4)
Other	2 (8)
Hispanic ethnicity, *n* (%)	6 (24)
Sexual identity	
Heterosexual or straight, *n* (%)	23 (92)
LGBTQIA +	1 (4)
Prefer not to answer	1 (4)
Partnered relationship status, *n* (%)	23 (92)
Education	
High school, some college and college graduate, *n* (%)	11 (44)
Post-graduate degree	14 (56)
Employed full-time, *n* (%)	20 (80)
Income	
< $99,999, *n* (%)	2 (8)
$100,000 to $149,999	2 (8)
$150,000–$249,999	12 (48)
> $250,000	9 (36)
***Infertility history***	
Infertility diagnosis, *n* (%)	
Male factor	7 (28)
Tubal factor	8 (32)
Endometriosis	1 (4)
Recurrent pregnancy loss	2 (8)
Advanced maternal age	4 (16)
Other	4 (16)
Years of infertility, median (IQR)	24.0 (12.0–48.0)
Prior IVF cycles, *n* (%)	
0	16 (64)
1	7 (28)
2 or more	2 (8)
***Baseline mood and quality of life measures***	
Depression (PHQ-9), mean (SD)	6.0 ± 4.55
Sleep disturbance symptoms (PHQ-9 item3), mean (SD)	0.8 ± 0.8
Clinically significant depression score (%)	6 (24)
Anxiety (GAD-7), mean (SD)	6.8 ± 4.9
Clinically significant anxiety score (%)	6 (24)
FertiQOL total score, mean (SD)	61.3 ± 15.5
Emotional subscale, mean (SD)	11.7 ± 3.8
Mind–Body subscale, mean (SD)	15.6 ± 3.8
Relational subscale, mean (SD)	16.0 ± 4.9
Social subscale, mean (SD)	14.7 ± 5.0
Social Readjustment Rating Scale (SRRS) total score, mean (SD)	6.5 ± 4.6
SRRS interpersonal life subscale, mean (SD)	1.5 ± 1.7
SRRS professional life subscale, mean (SD)	1.8 ± 1.6
SRRS home life subscale, mean (SD)	0.8 ± 0.5
SRRS identity and habits subscale, mean (SD)	2.3 ± 1.9

## Sample size

The recruitment of 26 participants was based on feasibility over the defined study period. The mean follicular phase length was 15.2 (SD 5.4) days. This sample size is powered to detect a 1.0 standard deviation difference in the mean number of days in the follicular phase of females who are more stressed versus less stressed (alpha = 0.05, power = 80%).

## Data analysis

Participant and cycle characteristics were described and summarized as proportions, mean (standard deviation), and median (interquartile range) as appropriate.

Functional principal component analysis (FPCA) was conducted to capture variability in daily stress measures among participants and to group participants by similar patterns. Guided by the proportion of variability explained by the top principal components (PC), the top two PCs were ascertained to summarize the major trends in change from the pooled mean absolute stress across participants.

Regression analysis followed with absolute stress measures or scores for PC1 and PC2 as the exposure with priming and COS cycle outcomes. Covariates and potential confounders for inclusion were initially selected based on prior studies. Models were adjusted for covariates in Table 1. Covariates exhibiting high multicollinearity were identified and excluded to avoid redundancy. The variable selection process was performed with least absolute shrinkage and selection operator (LASSO). For each outcome variable, leave-one-out cross-validation was used to ascertain the best regularization hyperparameter that produces the minimum cross-validation error with an exception of the case where regression separation issue occurred. The final model for the adjusted models included variables with a non-zero coefficient selected from LASSO for each outcome variable.

To explore if timing of stress in the early-, mid-, or late-follicular phase was associated with outcomes, each participant’s follicular phase was evenly divided into three intervals (early, mid, and late follicular), and daily mean, maximum, and minimum stress levels per participant were averaged within each interval and compared by outcome via regression analysis. To compare perceived stress between priming and COS cycles, paired *t*-tests were used.

## Results

From June to November 2023, 93 individuals were screened, 32 were eligible, 26 consented, and 25 participants completed the study. This cohort had an average age of 36 years (SD 3.6). A majority had a normal BMI (60%), were White (64%) and non-Hispanic (76%), identified as heterosexual (92%), and worked full-time (80%) (Table 1). The most common IVF indications were tubal and male factors (32% and 28%, respectively). Nearly one quarter of participants met criteria for clinically significant depressive symptoms and anxiety. FERTIQOL scores were moderately high, mean 61.3 (SD 15.5).

Text message response rates in the priming and stimulation cycles (from stimulation start to retrieval) were 55.6% and 75.7% per participant, respectively. The response rate for daily urine hormone measures testing during the priming month was 90.6%.

Priming and COS cycle characteristics and outcomes are summarized in Table 2. All 25 participants ovulated in the priming cycle after a median (IQR) of 14 (11–18) days in the follicular phase. Following COS, ten participants (40%) met criteria for the composite poor outcome.

**Table 2:** Priming and COS cycle characteristics and outcomes

Priming cycle	
Ovulation, *n* (%)	25 (100%)
Follicular phase length	
Median (IQR)	14.0 (11.0–18.0)
Urine LH at surge (mIU/mL), median (IQR)	20.5 (11.4–29.7)
**COS cycle**	
Stimulation days, mean (SD)*	10.5 (2.2)
Total gonadotropin dose (units), mean (SD)*	5137 (2615)
Peak estradiol (pg/mL), mean (SD)*	2977 (1481)
Composite poor outcome, *n* (%)	10 (40.0)
Cycle cancellation due to poor response, *n* (%)	3 (18.8)
Oocytes, mean (SD)** ≤ 4, *n* (%) > 4, *n* (%)	13.9 (6.9) 2 (9.1) 20 (90.9)
Mature oocytes, mean (SD)** ≤ 4, *n* (%) > 4, *n* (%)	10.8 (5.2) 2 (9.1) 20 (90.9)
Blastocyst embryos, mean (SD)** No blastocyst, *n* (%)	4.8 (3.2) 1 (4.5)
No euploid embryo, *n* (%)**	4 (18.2)
Embryo transfer outcomes***	
Clinical pregnancy following first transfer, *n* (%)	12 (66.7)
Live birth following first transfer, *n* (%)	12 (66.7)

*Among cycles with a retrieval (*n* = 22)

**Among cycles with an embryo transfer (*n* = 18)

***Clinical pregnancy and live birth outcomes were assessed only among participants undergoing embryo transfer. Outcomes were not applicable for seven participants (28%) who did not undergo embryo transfer

## Perceived stress and priming cycle outcomes

**Fig. 2:**
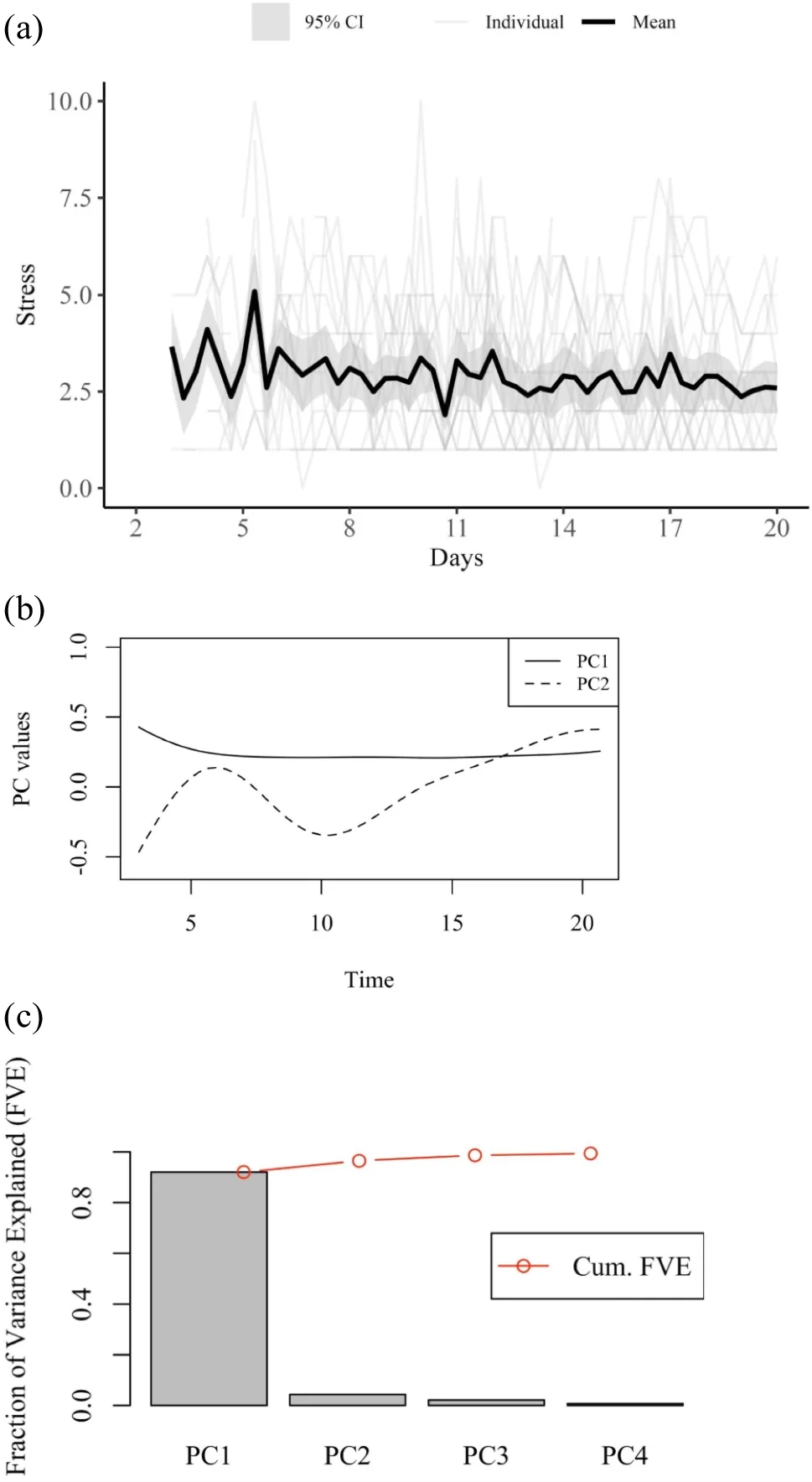
Priming cycle stress patterns. **a** Variability of mean stress of all participants with 95% confidence interval (*n* = 25) and individual participant trajectories. **b** Functional principal component curves (PC1 and PC2) capturing the two largest trends in variability in pooled mean stress among participants. **c** Fraction of variance explained by each principal component with cumulative fraction of variance explained overlay

Participant trajectories showed daily variability with approaching ovulation (Fig. 2a). Overall, stress decreased over the follicular phase. FPCA revealed two principal components that described a combined 96.5% (PC1 92.0% + PC2 4.5%) of variability in the pattern of stress (Fig. 2b, c). PC1 was the predominant principal component and demonstrates stress reducing over the early follicular phase then leveling out. PC2 captured a distinct stress peak around day 6, followed by a gradual increase over the mid- to late-follicular phase.

Regardless of stress pattern, all participants ovulated. There was no association between principal components and follicular phase length (*β*_PC1_ − 0.28 [95% CI − 0.71, 0.16], *β*_PC2_ − 0.74 [95% CI − 2.17, 0.68]).

PC1 was not associated with peak LH levels (*β* − 0.33 [95% CI − 1.1, 0.43]). Unadjusted linear regression demonstrated a negative association between peak LH level and PC2 (*β* − 2.64, 95% CI − 4.89 to − 0.39, *p* = 0.024), indicating that individuals with stress patterns more similar to PC2 had lower peak LH levels. Following inclusion of additional confounders and LASSO-based variable selection, this association remained significant (*β* − 2.52, 95% CI − 4.14 to − 0.90, *p* = 0.006). 

To explore the timing hypothesis that LH and follicular phase length could be associated with stress in specific parts of the follicular phase, we grouped stress into the first, second, and final third of the follicular phase and found higher minimal stress in the middle third was significantly associated with lower peak LH only in unadjusted analysis (*β* − 5.31 [95% CI − 9.75, − 0.88, *p*_unadjusted_ = 0.02]). Stress levels of these intervals were not related to follicular phase length.

## Perceived stress and COS outcomes

Following gonadotropin stimulation (mean days [SD] 10.5 [2.2]), mean oocytes retrieved and blastocyst embryos derived were 13.9 (SD 6.9) and 4.8 (SD 3.2), respectively (Table 2). There were ten participants (40%) who experienced the primary composite outcome of cycle cancellation, fewer than four oocytes retrieved, no blastocyst embryo development, or no euploid blastocyst.

**Fig. 3:**
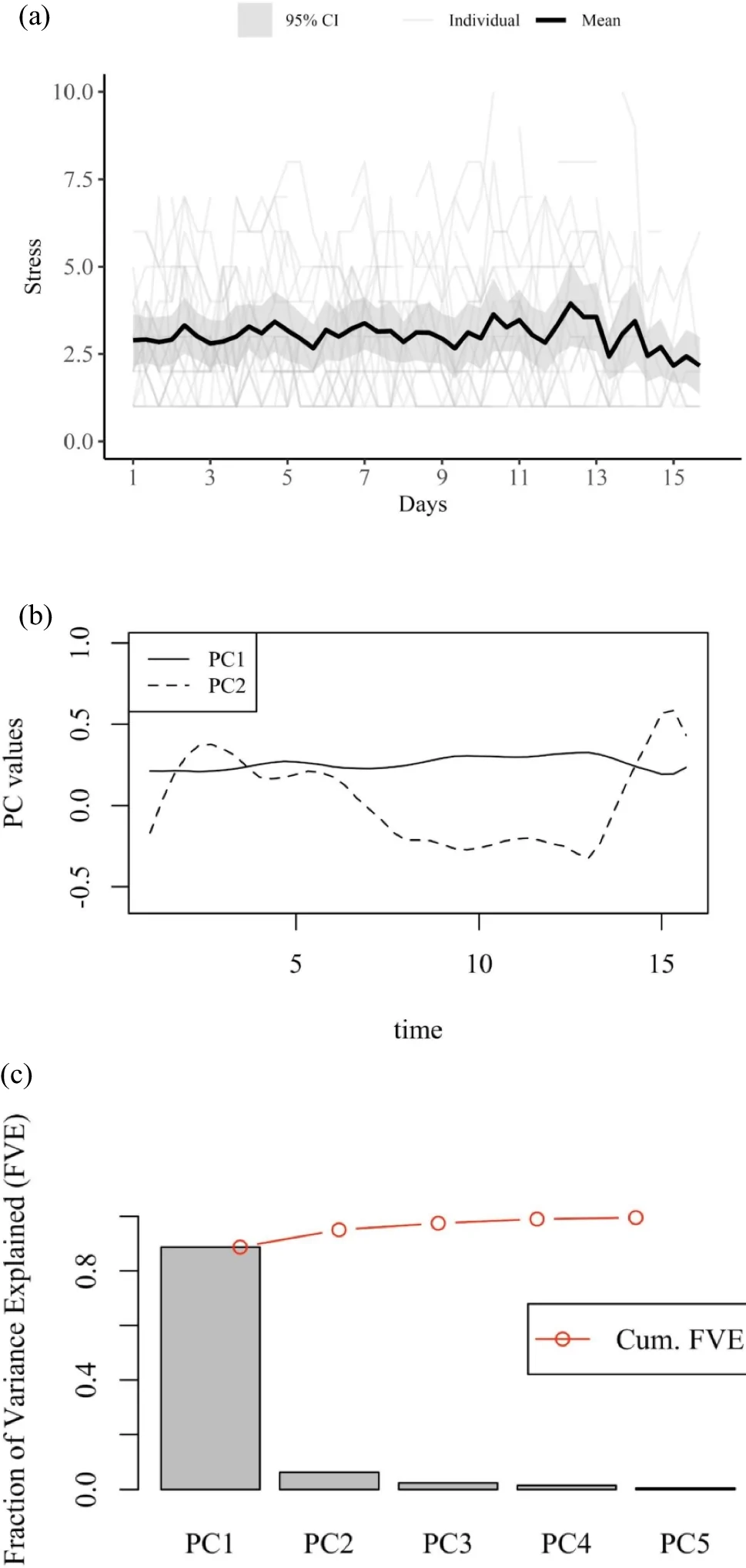
Controlled ovarian stimulation cycle stress patterns. **a** Variability of mean stress of all participants with 95% confidence interval (*n* = 25) and individual participant trajectories. **b** Functional principal component curves (PC1 and PC2) capturing the two largest trends in variability in pooled mean stress among participants. **c** Fraction of variance explained by each principal component with cumulative fraction of variance explained overlay

During COS, daily variability in stress was observed largely between 1 and 5 on the 10-point scale (Fig. 3a). FPCA revealed two principal components that described combined 95.0% (PC1 88.7% + PC2 6.3%) of the pattern of stress (Fig. 3b, c). Similar to the priming cycle, PC1 is the predominant principal component and was stable throughout COS. In contrast, PC2 captures a distinct trough in stress levels in the mid to late portions of COS.

Unadjusted linear and logistic regression showed no association between PC1, PC2, and the composite outcome or length of stimulation. Exploratory analyses evaluating clinical pregnancy and live birth outcomes following embryo transfer similarly demonstrated no significant associations with PC1 or PC2 of either cycle (data NS). Stress timing variables were likewise not associated with clinical pregnancy or live birth outcomes.

To explore the timing hypothesis, early-, mid-, and late-COS stress were not associated with the composite outcome or length of stimulation (data not shown).

## Perceived stress in priming vs. COS cycles

**Fig. 4:**
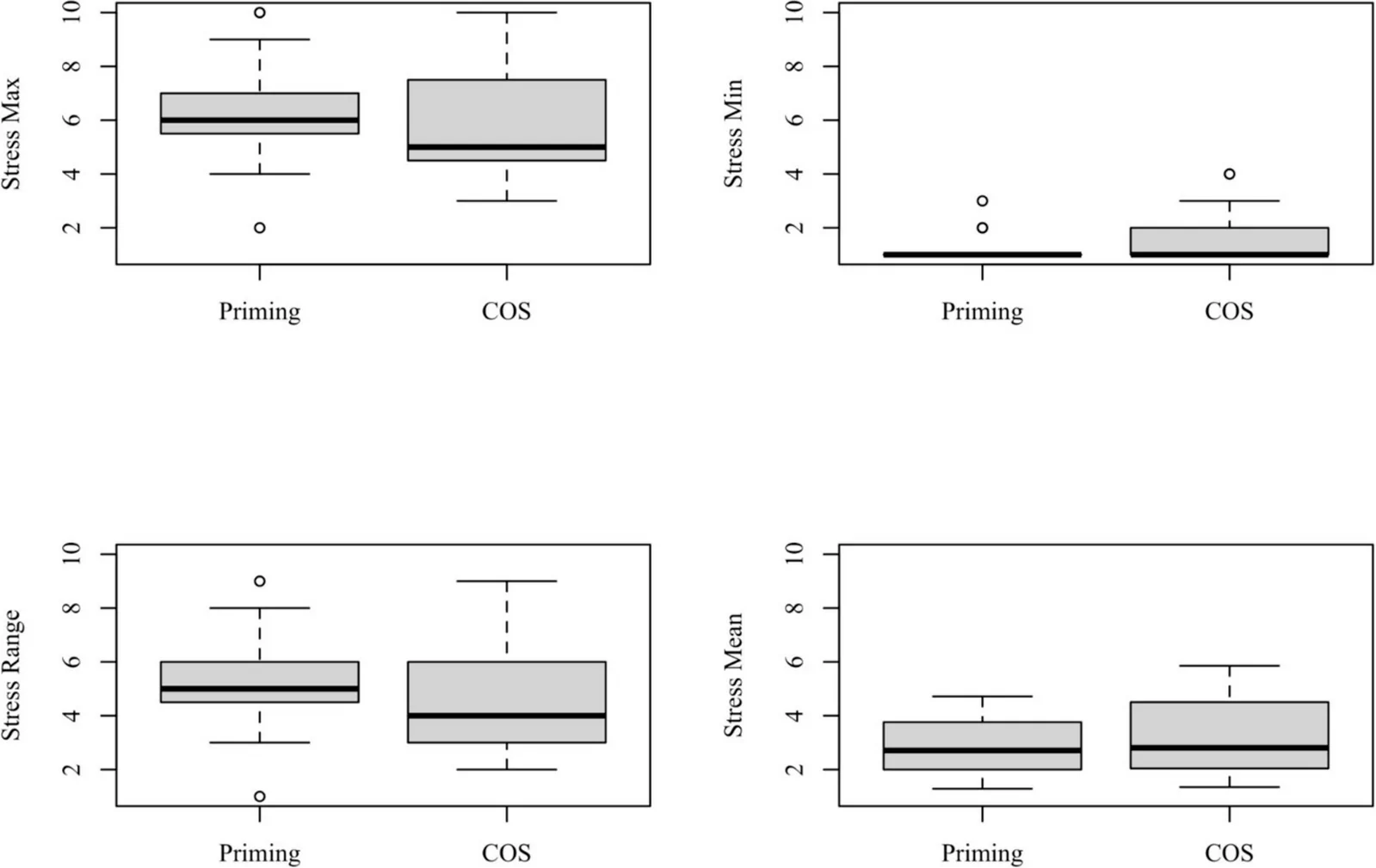
Comparison of perceived stress between priming and controlled ovarian stimulation cycles shown as box-and-whisker plots of participant-level daily summary measures (maximum stress, minimum stress, stress range, and mean stress) with boxes representing the interquartile range, center line the median, whiskers extending to 1.5 × interquartile range, and points indicating outliers

Paired analysis showed that absolute stress levels were similar between priming and COS cycles. Only minimal stress, the lowest stress level reported daily per participant, was on average lower in the priming cycle (*p* < 0.05) (Fig. 4).

## Discussion

This prospective cohort study leveraged EMA to phenotype patterns of stress perceived by females with infertility undergoing IVF across a natural and a COS cycle. On feasibility of ascertaining daily stress from this population, participants completed a majority of assessments. Data show a predominant pattern of moderate levels of stress that is largely stable over both follicular phases. In assessing the relationship between perceived stress and fertility, patterns of stress were not related to primary outcomes of ovulation, follicular phase length, and the composite poor outcome following COS, though LH levels were inversely related to an identified stress pattern. These findings support that perceived stress may be associated with subtle effects on HPO physiology without impacting primary clinical endpoints.

The present study used real-time, longitudinal measurement of perceived stress with EMA via text messaging, enabling capture of fluctuations across both natural and COS cycles. This approach presents a tool to augment available measures of perceived stress in people with infertility. Validated instruments to measure stress include the Perceived Stress Scale (four or 10 items to reflect stress over the past month) and the List of Recent Events (71 items) broadly, and specific to infertility, the COMPI Fertility Problem Stress Scale (14 items), Fertility Problem Inventory (46 items), and the Infertility-Related Stress Scale (12 items). However, these instruments were not designed to be administered repeatedly in short time intervals to enable measuring changes and patterns of stress [19–21]. Previously, summary data across studies of stress and IVF outcomes have suggested a small but significant negative correlation [22–26]. Thus, we were motivated to undertake deeper phenotyping and advanced statistical methods to summarize stress patterns. By applying FPCA to longitudinal stress data, we characterized stress patterns that extend beyond a binary or percentile classification of psychosocial stress [10], enabling decomposition of complex patterns in functional data to show temporal dynamics. While 90% of the stress pattern in both the priming and COS cycles was explained by one principal component that was largely stable over the observed periods, secondary patterns emerged showing temporal fluctuations.

Our novel approach supports no clinically meaningful impact of day-to-day perceived stress on fertility outcomes in people undergoing IVF. Additional analyses incorporating additional psychosocial and lifestyle confounders yielded findings consistent with the primary analyses, supporting the robustness of the observed null associations between perceived stress patterns and major IVF outcomes, including clinical pregnancy and live birth. Whether perceived stress is associated with fertility outcomes in people undergoing IVF is controversial, in part due to observational studies that cannot fully control for confounding [4, 27, 28]. In adjusted analysis, models did account for a series of known confounders such as body size. It is also possible that the measure of perceived stress is not specific to infertility-related stress; thus, no correlation was observed.

Anovulation was not observed in this cohort, which is likely due to accurate, daily at-home measures. We used a lateral flow-assay-based home fertility monitor to enable daily, simultaneous detection of urinary PdG, LH, and E3G. This monitor defines ovulation as > 2.75-fold PdG rise within three days after the LH surge (AUC 0.981) [9, 29, 30]. More recently, EAGeR trial participants used daily ovulation monitoring that assessed only LH and collected intermittent urine samples to measure LH, E3G, and PdG. This approach yielded ~ 12% risk of anovulatory cycles, which might have been even lower had PdG been measured daily [31].

Building on prior work examining perceived stress and fertility, we explored whether timing of stress during different stages of the menstrual cycle may result in different cycle aberrations and COS outcomes. One proposed mechanism suggested from preclinical studies is that stress-induced elevations in glucocorticoids and CRH exert negative feedback on hypothalamic kisspeptin neurons, key stimulators of the reproductive neuroendocrine axis. Kisspeptin neurons in the AVPV brain region are responsible for the preovulatory surge in GnRH release, which leads to the LH surge that triggers ovulation, while a separate population of kisspeptin (KNDy) neurons in the arcuate nucleus regulates pulsatile GnRH and LH release. Psychosocial stress can impair both LH pulses and the preovulatory LH surge, as shown in human and animal studies [32, 33]. These distinct functions of the two kisspeptin populations suggest that the timing of stress during different stages of the menstrual cycle may result in different cycle aberrations, such as impairments in surge versus pulsatile LH release.

Stress occurring during the early- to mid-follicular phase may preferentially reduce activity of KNDy neurons and decrease pulsatile release of GnRH and LH, prolonging the follicular phase [12]. Stress present during the late follicular phase may negatively regulate AVPV kisspeptin neurons, thus inhibiting GnRH and LH surges and preventing or delaying ovulation [13]. This mechanism aligns with the lower peak LH levels observed in our cohort. A priori, we hypothesized that stress in the early and mid-follicular phases would reduce neuronal activity of KNDy neurons, decrease pulsatile release of GnRH and LH, and prolong the follicular phase, but we did not observe this association. We also hypothesized that significant stress during the late follicular phase would negatively impact AVPV kisspeptin neurons and delay or prevent the LH surge, but we did not observe anovulation. As the timing hypotheses were exploratory and not powered, the observed signal warrants further investigation.

A major strength of this study lies in its methodological innovations. EMA allowed for real-time, continuous measurement of stress over time, providing insights into stress patterns that would otherwise be missed by traditional, discrete assessments. The use of current, accurate lateral flow assays to detect the temporal patterning of both LH and PdG at home enables adherence to measurement, mitigates the risk of misclassification, and demonstrates an important cost-effective advancement for ovulation studies in fertility research. Finally, the FPCA approach enabled the decomposition of complex stress patterns in functional data, revealing temporal dynamics that are often overlooked by traditional measures.

This study had several limitations. Although the sample size was sufficient to detect large effect sizes, it was not powered for smaller effects or stratified subgroup analyses. We did not directly measure physiologic stress markers such as cortisol, but there are no gold-standard objective measures of stress. Perceived stress was not specific to infertility, but selected to enable daily capture and model patterns [10, 15]. It is possible that we recruited a lower stress population that may not have reached a threshold likely to impair reproductive physiology. Mean perceived stress scores fell within the low-to-moderate range (approximately 2–5 on a 10-point EMA scale), and it is plausible that our participants’ physiological stress burden was limited.

In conclusion, while perceived stress may subtly influence HPO function, it does not appear to significantly impact fertility outcomes for patients experiencing mild-to-moderate stress levels as they undergo IVF. These findings are reassuring for this population, as they suggest that perceived stress is largely stable and does not compromise primary outcomes such as ovulation.

## Data Availability

The datasets generated and/or analyzed during the current study are not publicly available due to ethical and privacy restrictions related to human participant data, but are available from the corresponding author on reasonable request and with appropriate institutional approvals.
